# A Study and Analysis of the Relationship between Visual—Auditory Logos and Consumer Behavior

**DOI:** 10.3390/bs13070613

**Published:** 2023-07-24

**Authors:** Hui Li, Junping Xu, Meichen Fang, Lingzi Tang, Younghwan Pan

**Affiliations:** 1 College of Fine Arts, Guangxi Normal University, Guilin 541006, China; meishaonuhui@gxnu.edu.cn; 2 Department of Smart Experience Design, Kookmin University, Seoul 02707, Republic of Korea; xjp1110@kookmin.ac.kr (J.X.); fangmeichen@kookmin.ac.kr (M.F.); 3 School of Humanities, Arts and Design, Guangxi University of Science and Technology, Liuzhou 545006, China; tanglingzi@gxust.edu.cn

**Keywords:** visual and auditory logo, interactive relationship, consumer perception, consumer behavior

## Abstract

Given enterprises' participation in market competition and the development of sensory marketing, in addition to the traditional visual identity, some enterprises gradually begin to pay attention to auditory and then introduce sound design when designing logos. Audio-visual stimulation and media innovation are committed to creating positive attitudes among consumers. This study constructs a model of visual and auditory interactive relationships with consumer behavior using the SOR model. It tests the conceptual model and checks the hypotheses proposed in the study. This study summarizes and contributes to the visual and auditory interactive relationship between information integration, information synergy, mutual competition, and matching degree. It further proposes the influence of purchase intention and consumer support on consumer behavior of perceived brand perception, credibility, and quality perception. The results and highlights ensure brand identities reflect a significant positive result through consumer behavior. In this paper, we collected questionnaires from a random sample of 1407 respondents. We used regression analysis to test the association between visual and auditory interactive relationships as well as consumer behavior. We further verified the mediating role of consumer perception variables. Adding audiovisual logo design to the marketing process can be an effective way for companies and brands to attract customers and increase their support and purchase intentions.

## 1. Introduction

**Figure 1:**
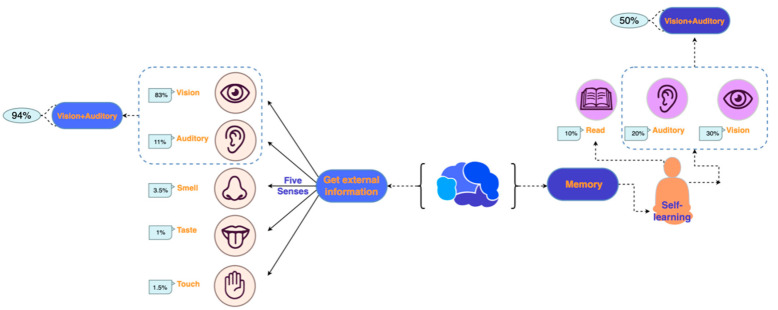
The proportion of external information received by the human brain through the five senses.

With the further development of marketing research and the gradual introduction of psychological research results, sensory marketing has become familiar to the public. What consumers buy is no longer the product itself, but also the various sensory stimuli and experiences in the consumption process. Krishna [1,2] proposed a complete theory of sensory marketing, which means that sensory marketing is a marketing approach that incorporates the five sensory experiences of consumers (touch, sight, taste, smell, and auditory) and influences their perception, judgment, and behavior. All the senses receive different amounts of information. Treicher, a famous experimental psychologist, showed that humans get external information through the five senses. Of these, 1% comes from taste, 1.5% from touch, 3.5% from smell, 11% from auditory, 83% from vision, and 94% from sight and sound combined. Additionally, he outlined the hypothesis that most people can remember 10% of their reading content, 20% of their auditory content, 30% of their visual content, and 50% of their visual and auditory content. This shows that the combination of audio–visual design in logo design provides an advantage for the interaction between corporate brands and consumers [3], as shown in Figure 1.

Visual and auditory senses play a crucial role in receiving information and remembering content [4], which has recently attracted the attention of the field of corporate brand theory and practice. In addition to the original visual design part, some enterprises also began to introduce auditory design in the design of corporate brand logos and strive to let consumers receive brand and product information through visual and auditory. In addition to the legislative protection of traditional visual logos, international protection of auditory logos has also entered the legislative field. This has laid a legal foundation for auditory logo protection. For example, international intellectual property conventions such as the Agreement on Trade-Related Aspects of Intellectual Property Rights (TRIPS), the Paris Convention, the Madrid Agreement, and the Trademark Registration Treaty support auditory trademarks.

Scott et al. [5] demonstrated how, despite their short display intervals, sonic logos identify with users’ sentiments and viewpoints. This was primarily associated with long-running campaigns and ad campaigns. The directing efforts of Sonic brand logo promotion in promotion (starting instead of announcing) with the interceding function of feeling exposed as a business and noticed were likewise investigated. The development currently, sonic symbol testing is accomplished through a pair of tests, each comparing nine theories. A preparatory test is further completed to make identical symmetrical sonic trademarks, which are ultimately utilized in both trials. An advertisement with a happy Sonic logo received higher attitude scores from participants than an advertisement with an unhappy Sonic logo. The Sonic logo placement within the advertisement moderates these consumer attitudes. It is mediated by the intense feeling evoked upon the introduction of the company and promotion. The situation created increased favorable user perspectives for a miserable sound logo at the beginning and a cheerful sound logo at the end of each ad. An exploration of restricted sonic logos is developed in this study. There is a possibility that a sonic mark may possess the same close-to-home qualities as lengthy structure music during the brevity openness phase. A recent version of this was saved for ambient sound in the promotion. Moreover, by revealing the interceding involvement with feeling following openness in respect of the trademark and commercial, as demonstrated that there are a few sound marking components that can help form feeling with these shopper mentality affections for brand names. Lastly, changing where the Sonic logo is placed can change how customers feel about the advertisement and the brand.

The contribution of the theoretical study is to demonstrate that it is possible to enhance the identity of a brand with the use of the most effective sound logos. Logos are usually associated with visual design, but sound can also serve as an important identifier. The use of sonic logos is, therefore, becoming increasingly popular among brands. Soundbites are often the best sonic logos, lasting no more than a few seconds at most. The use of audio-only media such as radio, podcasts, or apps like Spotify may substitute the visual logo for use with audio-only media, strengthening overall brand recognition. Sound logos are usually trademarked by the company, and they need to meet many of the same requirements as visual logos, such as being simple, instantly recognizable, and provoking emotions.

Sensory elements can have a non-conscious impact on consumer judgments and purchase behavior and are an essential component of both offline and online retail stores. Customers’ shopping experiences and purchasing habits are influenced by ambient factors like scent, lighting, and music in offline settings. For online e-commerce outlets, tangible variables connected to a variety of products, examples, and designs can affect shopper conduct. Because they influence behaviors unconsciously and are relatively simple to change, sensory elements have significant managerial relevance. For instance, altering the display pattern for an online store or the music or lighting in a physical retail store requires no effort [6].

This review of mental imagery research encourages additional research into sensory imagery. The review is organized around a conceptual framework that focuses on how mental imagery is formed. It also focuses on how mental imagery is elicited and elaborated, how sensory imagery is multimodal, and how mental imagery affects consumer behavior. This conceptual framework inspires novel interdisciplinary research concepts by providing fresh perspectives on previous findings. In the final section of the review, additional unexplored opportunities are presented in addition to future research directions [7].

Although virtual merchandising has established itself as an indispensable 21st-century necessity, people who are sighted or with low perception nevertheless have serious difficulties interacting with it despite insufficient picture depictions and the powerlessness to channel a lot of data utilizing screen perusers. As a solution to those problems, Redo was proposed as a framework for intuitive data recovery that relies on client surveys. An analysis-based solution and communication can be facilitated via Redesign on a rebuilt product page. From our meeting, we distinguished four fundamental perspectives that are essential for BLV clients to grasp the ocular exterior of an item. We developed annotated procedures to retrieve code extracts depending on the findings. These rules generated image descriptions and user queries. An evaluation was conducted on eight BLV users to determine whether Revamp helped them locate key information efficiently and presented constructive expression in return for thoughtful creation forms [8].

As a visual aid, logos help businesses convey their identity and attract customers’ attention. Even though the significance of logos and their widespread use is evident, the practice of logo writing remains divided. As a result, this article attempts to provide a general research framework by conducting a thorough review of the existing literature on logos. In particular, the study sorts there have been 124 scholarly studies in management journals over the last 30 years. These studies are grouped into six main subject areas: hypothetical establishments, logo configuration/overhaul, fundamental logo components, extra logo components, results of the implementation of logos, and pragmatic utilizations of symbol application. Finally, it provides practitioners with guidelines for managing logos for their businesses and academics with directions for research [9].

For a green product, the logo should fit well with the brand character. A logo’s shape has a significant impact on consumers’ perceptions and preferences since it is a basic element of the logo. Brand logos are often asked whether they should be angular or rounded. In this case, which shape should be used for a green product’s logo, angular or rounded? As part of previous studies, researchers examined how the shape (angular or rounded) of brand logos affects consumers’ perceptions of color, investigated the functional mechanisms contributing to this association, and explained the results based on embodied cognition theories. The situation is still devoid of comprehensive and in-depth analysis of consumer interaction between various sensory factors and their impact on consumers’ brand logos. Moreover, there is a lack of corresponding theoretical support applied to brand identity conceptualization and marketing practice. There is an urgent need to conduct further research based on sensory marketing theory achievements. This is to clarify the interaction of various sensory factors and their impact on consumer behavior. Therefore, this paper selects the influence through the interaction between the two senses of sight and hearing that receive the most external information on consumer behavior as the research object and takes consumer perception as the mediating variable to explore the impact of the interaction between visual and audio brand logos affecting consumer behavior, as well as the role of consumer perception in determining the effect of the interplay between visual or auditory brand logos and consumer behavior.

## 2. Literature Review

In daily life, people often receive stimuli in sequence starting with multiple minds at the identical instance. The brain controls behavioral responses and allocates attention by integrating stimulus information from multiple sensory organs [10]. Moreover, different brain regions use different strategies to interact, integrate information, and establish a unified perception [11].

It is well known that the role of vision and auditory in the brain is not consistent. Colavita [12] found a visual dominance effect when subjects were asked to respond to visual and auditory stimuli. According to Welch and Warren [13], the visual dominance effect occurs because visual perception is essential for perceiving, while temporal perception is vital for auditory perception. In the study of the interaction effect between vision and auditory, Shams et al. [14] and Marakshina [15] confirmed that vision and auditory interact and used brain imaging data to compare spatial and temporal attention. Calvert et al. [16] studied the integrative effects of vision hearing, finding that simultaneous implementation of interactive audio–visual stimuli had a higher activation intensity on specific sensory cortices than single channel stimuli. Mishra et al. [17] further examined visual–auditory integration by magnetic resonance imaging (fMRI), validating the CALVERT findings. Yordanova et al. [18] and McDonald et al. [19] studied the visual–auditory synergy. Hein et al. [20] and Koelewijn et al. [21], on the other hand, studied visual and auditory mutual competition for attentional resources. However, there are certain conditions for audio–visual integration effects. Meredith et al. [22] and Navarra et al. [23] studied the temporal interval problem of visual and auditory information matching and found that the audio–visual integration effect disappears when the interval increases to 1000 ms. Iwamiya [24] investigated the matching degree of visual and auditory information and tried to score it.

The impact of store atmosphere on the in-store experience has been studied in previous studies. Alternatively, sensory marketing and brand experience have been found to provide better consumer experiences. Thus, the purpose of this paper is to extend the scope of this study by studying the causal relationship between sensory marketing cues and brand experience on emotional attachment and subsequent brand loyalty. Moreover, sensory marketing cues and brand experiences also contribute to consumers’ emotional attachment to luxury brands [25].

Researchers Scott et al. [5], show how short-exposure time sonic logos can have the same emotional properties as long-form background music, previously used only in advertising for background music. It is also shown that these short audio branding elements can help shape consumer attitudes toward brands by uncovering the mediation relationship of emotion after exposure to a brand and advertisement. As a final point, repositioning the Sonic logo can boost consumer attitudes toward the brand and advertisement. Consumer behavior and the interaction effect of Visual and Auditory Logos are explored in this study.

In a retail environment, atmospheric cues may enhance consumer behavior, but they can also overwhelm consumers due to too much stimulation [26]. It is, therefore, chosen for this study to study the influence of sight and hearing on consumer behavior that occurs as a result of interaction between those two senses. The mediating variable is consumer perception, which investigates the influence of visual and audio brand logos on consumer behavior as well as the implications of consumer interpretation on consumer behavior when interacting between visual and auditory brand logos.

## 3. Materials and Methods

### 3.1. Research Model

**Figure 2:**
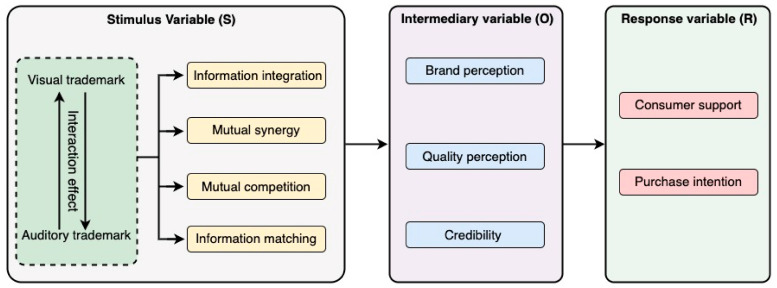
Structure diagram of the research model.

Using the keywords “sensory marketing”, “visual logo”, “auditory logo”, and “social media” on CNKI and Google Scholar Interaction search the literature for the interaction effect of the visual logo and the auditory logo. Using the theory of sensory marketing and the theory of audio–visual interaction stimulation and based on An Xingwei et al. [27] research results, the questionnaire was compiled. Additionally, a study was carried out on the integration of related data, mutual synergy, mutual competition, and matching degree of audio–visual interaction stimulus in brand logos, along with social media’s media role. Russell and Mehrabian [28] proposed the SOR model, which is a “stimulus-organism-response” model, in which stimulus (S) refers to the driving force that may influence consumers’ cognitive processes and is an external factor related to consumers’ decision-making behavior. Organism (O) involves the processes and structures within the individual between the stimulus and the final behavior and response, which consists of human emotions and cognition. As a result of the consumer’s response (R), a consumer’s final attitude or behavior is described by Gatautis et al. [29]. Specifically, this study took the four aspects of the interaction effect that affect vision and auditory as stimulus variables, the impact of information integration, mutual synergy, mutual competition, and information matching, took consumer perception, quality perception, and credibility of the brand as mediating variables, and consumer perception of brand and purchase intention as response variables. Figure 2 shows the research model.

### 3.2. Hypothesis

Seifipour and Mokhtarian [30] investigated the effect of the interrelationship between trade names and logos on customer attitudes and behavioral loyalty. They considered corporate relationship excellence’s moderating impact. Ng’etich [31] argued that the study reveals a significant relationship between advertising and consumer purchase decisions and that marketers can effectively use advertising to influence consumers to purchase their products/services. Guoqi et al. [32] found that free-surfing consumers enhance purchase intention by processing ad images and logos to generate positive information effectiveness and purchase demand consumers inhibit purchase intention by processing banner headlines to generate negative information effectiveness.

Based on this, the following hypothesis can be obtained:

**H1.**  *The interaction effect of audio-visual logos is positively correlated with consumer behavior*.

**H1a.**  *Information integration of audio–visual logos is positively correlated with consumer behavior*.

**H1b.**  *Information synergy of audio–visual logos is positively correlated with consumer behavior*.

**H1c.**  *Mutual competition of audio–visual logos is positively correlated with consumer behavior*.

**H1d.**  *Information matching of audio–visual logos is positively correlated with consumer behavior*.

Sichtmann and Diamantopoulos [33] found that brand image has a significant effect on consumer purchase behavior. Researchers at Cai and Aguilar [34] found that consumers are more willing to purchase a product when their perceived evaluation of the brand image is high. Tu et al. [35] argued that consumers’ brand perceptions, including the degree of involvement in the product or purchase decision, the perception of differences between brands, and the pursuit of hedonic consumption/utility consumption, all influence consumers’ purchase behavior.

Meanwhile, Calvo Porral and Levy-Mangin [36] concluded that credibility provides a major regulating function affecting shoppers’ loyalty to private-label foods. Lin and Lu [37] concluded that credibility is the presence of a notable uplifting impression affecting consumer decision-making preferences. Rubio et al. [38] have pointed out that customer loyalty and credibility are the key factors for retailers.

Grewal et al. [39] argue that brand perception quality impacts perceived value and thus positively influences customer buying behavior. Dodds et al. [40] also concluded that perception quality has a significant effect on perceived value and purchase intention. Moreover, Ratnam et al. [41], found that brand elements and brand perception quality have a significant impact on Cargill brand purchases.

Based on this, the following hypothesis can be obtained:

**H2.**  *Consumer perception plays a mediating role between the interactive effect of audio–visual logos and consumer behavior*.

**H2a.**  *Brand perception plays a mediating role between the interactive effect of audio–visual logos and consumer behavior*.

**H2b.**  *Credibility plays a mediating role between the interactive effect of audio–visual logos and consumer behavior*.

**H2c.**  *Quality perception variable plays a mediating role between the interactive effect of audio–visual logos and consumer behavior*.

**Figure 3:**
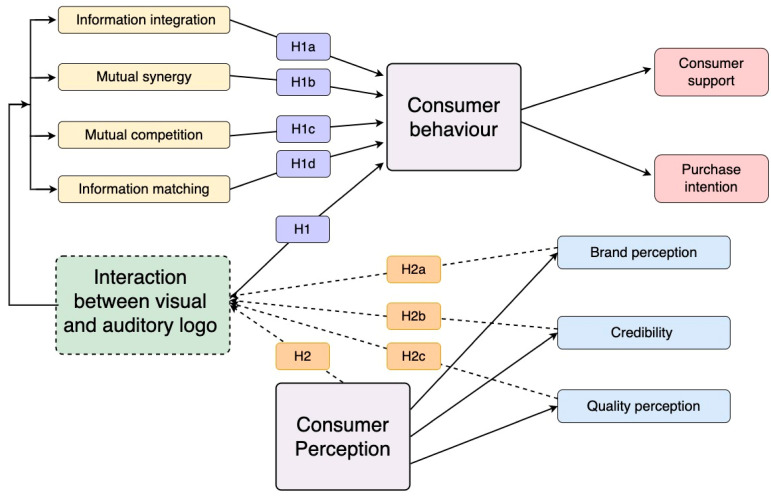
Hypothesis diagram between variables.

The research model is shown in Figure 3.

### 3.3. Method (Methodology)

#### 3.3.1. Data Survey

A questionnaire survey is conducted. In the process of compiling the questionnaire, variable questions were stimulated successively with (SQ) according to the S-O-R model. The intermediate variable (OQ) represents the consumer perception survey scale, while the response variable (RQ) represents the serial number.

The questionnaire was administered on a five-point Likert scale, with 1 indicating “totally disagree” and 5 indicating “totally agree”.

#### 3.3.2. Preparation of Questionnaires

For the interaction effect data of visual and auditory logos on stimulus variables, the author compiled a questionnaire on the interaction effect of auditory and visual logos. This was based on previous studies by Anderson [42]; Hinman et al. [43]; Vuoskoski et al. [44]; Fullwood et al. [45]; Pinto [46]; Vijayasarathy, [47] and Xiao et al. [48] and several scholars’ research results. Among them, the stimulus variables are 24 questions in four dimensions such as information integration, information synergy, mutual competition, and information matching. These questions are detailed in Table 1.

The consumer perception of the mediating variable includes three aspects. The first is the enterprise brand image data, which was compiled based on scholars such as Lafferty [49], as well as Bagozzi et al. [50]. It includes two dimensions, cognitive brand attitude and affective brand attitude, with six question items. The second type of data is information about the perceived quality of the company’s products. This data scale was developed from the scale used in the study by Zboja and Voorhees [51] and others. Third, for consumer quality perception, this paper adopts and modifies the scale used by Zhou et al. [52] in the 2007 study, which consists of three items. Table 2 shows the details.

For the data on consumer purchase intention and consumer support behavior as the response variable, the consumer support measurement scale was developed by Zeithaml et al. [53] and the consumer purchase intention scale prepared by Dodds and Dodds et al. [40] was used. Table 3 shows the details.

#### 3.3.3. Reliability and Validity Analysis of Scale

Regarding the reliability analysis of the scale, the most commonly used Cronbach’s alpha coefficient (Cronbach’s α) was chosen as the testing variable in this paper. Nunnally [54] says if Cronbach’s α is greater than 0.7, the scale has high reliability. From the reliability analysis measurements, the reliability test results of the six variables involved in this survey were all over 0.8. The sample reliability results indicate that the scale used in this study has high reliability. Table 4 shows the details.

Validity analysis examines the extent to which an indicator can measure the true nature of things, including content validity and structural validity. Based on previous relevant studies and combining the questions in this study, the scale was developed. It has a significant amount of content validity. Structural validity measures the degree of tightness between variables. KMO and Bartlett’s tests are commonly applied to test this scale’s validity. For the KMO test, factor analysis is appropriate for factors above 0.7. For the Bartlett test, if the significance is less than 0.05, factor analyses are possible. Using the results of validity testing, a reliability evaluation is conducted and the test results of the six variables involved in this survey, the KMO values are over 0.8, and the significance in the Bartlett Test of Sphericity is less than 0.05, which means that the data of this survey can be utilized for factor analysis. Table 5 shows details.

According to Supplementary Figure S1, our focus for the present will be on building a CFA (confirmatory factor analysis) model, which demonstrates how a well-fitting measurement model can be used to test hypotheses relating to the structural relationships between latent variables from the F1 Consumer Behavior variables associated with Brand perception, Credibility, and Quality perception, and according to Consumer perception variables, F2 is divided into Consumer purchase and Consumer support variables.

According to Supplementary Figure S2, A latent variable SEM approach is used to model the aggregate effects of common and rare variants in multiple potentially interesting factors. A model of the relationship between consumer behavior and consumer perception which may be associated with variables such as brand perception (0.878), credibility (0.833), and quality perception (0.859). Consumer support (0.819) is higher than consumer purchase (0.842).

## 4. Analysis of Data Survey Results

### 4.1. Studying the Interaction of Visuals and Sounds with Logos to Influence Consumer Behavior

A total of 1500 questionnaires were distributed during October 2022, and 1407 valid questionnaires were collected. In terms of gender, there were 714 males (50.75%) and 693 females (49.25%); in terms of age, they were mainly concentrated between 25 and 50 years old, with 83.29% of the sample in this age group; in terms of education, 558 (39.66%) had college education or less, 687 (48.83%) had undergraduate education, 124 (8.81%) had master’s degree, and 38 (2.7%) had doctoral degree; in terms of occupation, professional, and technical personnel and clerical personnel accounted for the largest proportion, accounting for 71.77%; in terms of living areas, 160 (11.37 percent) live in Metropolises with large populations of 5 million to 10 million, 602 (2.79 percent) live in the largest cities as measured by their size of 5 million to 10 million, 486 (34.54 percent) live in megacities among a people of one million to five million, 99 people (7.04%) lived in medium-sized cities (including county seats) with a population of 500,000 to 1 million, 45 people (3.2%) lived in there are few places with a community of smaller than 500,000, and 15 people (1.07%) lived in towns or rural areas.

To analyze the effect of audible and visual logos on consumer behavior, this study used SPSS23 software for hypothesis testing. Model 1 is a regression model with personal characteristics as control variables (gender, age, education, occupation, and region), audio–visual logo interactive relationship as an independent variable, and consumer behavior as the dependent variable; Model 2 is a regression model with the sub-variables of the audio–visual logo interactive relationship (audio–visual information integration, audio–visual information collaboration, audio–visual mutual competition, and audio–visual information matching) as the independent variable and consumer behavior as the dependent variable. Table 6 shows details.

In the regression model with the interactive relationship of audio–visual logos as the independent variable and consumer behavior as the dependent variable, the β series coefficient is 0.697, R-squared is 0.609, F is 363.949, with significance *p* < 0.01, and passes the regression test. X_0_ assumes the independent parameter and Y represents the dependent factor, and thus the following regression equation can be obtained: Y = 0.697 × 0 − 0.499.

Based on the results of this study, it can be seen that hypothesis H1 is true, and the audio–visual interaction effect leads to a positive change in customer behavior. The regression test was conducted with the four variables that make up the interaction effect of visual and auditory logos based on separate variables and consumer behavior as a reliant parameter. The outcome showed that the R-squared was 0.615 and the F was 248.267, with significance *p* < 0.01, and passed the regression test, and the regression coefficients were all positive. The β coefficients of audio–visual information integration, audio–visual information synergy, audio–visual mutual competition, and audio–visual matching degree were 0.138, 0.25, 0.137, and 0.176, respectively.

With X_1_, X_2_, X_3_, and X_4_ representing independent variables and Y representing dependent variables, the following regression equation can be obtained:

Y = 0.138X_1_ + 0.257X_2_ + 0.137X_3_ + 0.176X_4_ − 0.539.

This equation also shows that four variables, such as audio–visual information integration, audio–visual information synergy, audio–visual mutual competition, and audio–visual matching degree, all have a positive effect on consumer behavior. In summary, the audio–visual interaction effect has a positive effect on consumer behavior, so hypotheses H1a, H1b, H1c, and H1d are all true.

### 4.2. An Analysis of the Interaction Effect of Consumer Perception on Visual and Auditory Logos and the Mediating Effect of Consumer Behavior

Regression analysis was performed by SPSS23 software with the interactive effect variable of audiovisual and acoustic logos as a set of attributes, consumer behavior as a dependent variable, and consumer perception variables and three sub-variables as mediating variables, respectively. Regression tests produced the following results. Table 7 shows the details.

Taking the consumer perception variable as the intermediary variable: According to model 3 in the table, R-squared, F-value, and significance *p*, all passed the regression test. At the same time, it can be found that the absolute values of the regression coefficients of the independent variables are smaller than those before the introduction of the mediating variables, indicating that the mediating variables enhance the greater degree of impactful capturing impact on the visual and acoustic advertising interaction effect and consumer behavior. Hypotheses *H2, H2a, H2b,* and *H2c* are all true.

In this paper, we will develop a CFA (confirmatory factor analysis) model which shows how a well-fitting measurement model can be used to test hypotheses relating to structural relationships between latent variables from the F1 Consumer Behavior variables associated with Brand perception, Credibility, and Quality perception. F2 is divided into Consumer purchase and Consumer support variables based on Consumer perception variables.

In Supplementary Figure S2, common and rare variants are combined into an aggregate effect by using a latent variable SEM approach. An analysis of the relationship between consumer behavior and consumer perception may include variables such as brand perception (0.878), credibility (0.833), and quality perception (0.859). There is a greater difference between consumer support (0.819) and consumer purchase (0.842).

## 5. Discussion

Males made up 50.75% of the audience, according to the frequency analysis examining the combined contribution of visually appealing and acoustically appealing logos during consumer behavior. They were mostly between the ages of 25 and 50, with 83.29 percent of the sample falling into this age range; with regards to schooling, 39.66% had school training or less, 48.83% had undergrad instruction, 8.81% had a graduate degree, and 32.7% had doctoral certificate; as far as an occupation, expert, and specialized staff and administrative faculty represented the biggest extent, representing 71.77%; In terms of living areas, 11.37 percent live in megacities with a population of 5 million to 10 million, 2.79 percent live in major cities and regions with an inhabitant of 5 million to 10 million, 34.54 percent live in other top cities with a demographics of 1 million to around five million, 7.04 percent live in medium-sized cities (including county seats) with a population of 500,000 to 1 million, 3.2 percent live in a small city has fewer than 10,000 people 500,000, and 1.07% live in towns.

As well as, As per An examination of the collaboration impact of purchaser discernment on visual and hear-able logos and the intervening impact of buyer conduct it tends to be found that the surviving exploration in cooperation impact of shopper discernment on visual and hear-able logos and the intervening impact of customer conduct essentially centers around the elements and results of logos that affect buyer conduct, yet little inspected the plan of brand logos. There has been a connection between these findings and the study of Dodds et al. [40], According to the theories of absolute values, the regression coefficients of the independent variables are smaller than they were before the influencing variables were added. This indicates that the mediating variables have a more significant mediating effect on consumer behavior and the effect of interaction between visual and acoustic advertising. The hypotheses that resulted (*H2, H2a, H2b,* and *H2c*) are valid. We investigated the effect of consumer perception variables and verified their mediating role. According to the data, audio–visual logo design, which stimulates customers’ visual and auditory senses, can be one of the practical ways for businesses and brands to attract customers, increase customer support, and increase purchase intent during the marketing process. This has been validated by the previous study by Tu et al. [35]. Logos significantly influence consumer behavior.

A positive interaction effect between visual and auditory logos is necessary for consumer support. It can be seen from the research that there is a positive correlation between visual and auditory interaction effects and consumer behavior. In other words, the more favorable the visual and auditory interaction effect is, the more significant its impact on consumer behavior will be. It can be concluded that in the process of making and releasing a visual and auditory logo, an enterprise must pay attention to establishing a benign interaction effect. As a result, the two will not weaken or antagonize each other. For example, if the information of visual and auditory logos cannot enable consumers to form integrated corporate image information, or the two pieces of information cannot be coordinated, or mismatched, or there is too intense opposition, it may affect the actual effect of the logo, which is not conducive to small branches to obtain complete corporate information through visual and auditory logos and form purchase intention and support behavior.

Brand perception, credibility, and quality perception variables in consumer perception influence consumer behavior. As enterprises create and publish visual and auditory logos, they should think about how these processes relate to the brand, credibility, and quality of the products, as well as the results of the Calvo Porral and Levy-Mangin [36] study. They must also avoid the appearance of unfavorable content in the logo. Once there is content or information that is detrimental to the perception of corporate brand image, brand reputation, or product quality, it will certainly lead to the design and release of the logo cannot achieve the expected effect and even affect the enterprise’s sustainable development.

This study also has certain limitations that need to be remedied in future studies. They are mainly reflected in the following two aspects:

The scales used in this study all refer to previous research results and mature literature scales. The author has combined these research achievements with scales for the first time. This is especially the scale of the interaction effect at play between visual and audible logos studied here. While its size has passed the test of reliability and validity, it still needs further research and development. There is a lack of consideration of individual consumer characteristics in the research. According to research results, not only different types of consumers have significant differences in their perceptions of logos. In addition, consumers also have unique preferences for purchasing and supporting behaviors. Therefore, studying the perceptions and reactions of different types of consumers to visual and auditory logo interaction effects will help companies carry out their logo production and release activities more effectively, which is also the direction of future research.

## 6. Conclusions

For modern companies, designing and publishing visual and auditory logos is a crucial tool to expand the market. How to gain consumer recognition and support through the design and release of visual and auditory logos is a highly valuable research issue. In this study, a linear regression research method was used to conduct an in-depth study of consumer behavior in the context of the interaction effect of corporate visuals and auditory logos. In addition, this paper examines the mediating effects of consumer quality perception, consumer trust, and consumer brand perception on the interaction effect between visual and auditory logos and consumer behavior. The results show that the interaction effect of visual and auditory logos of companies has a significant positive effect on consumer behavior. The four variables that make up the interaction effect of visual and auditory logos, namely audiovisual information integration, audiovisual information synergy, audiovisual mutual competition, and audiovisual information matching, all change consumers’ behavior favorably. The conclusion of this study suggests that for future corporate logos, it is necessary to fully explore the interaction effect between the visual logo and auditory logo, promote information integration, information synergy, and information matching of visual and auditory logos and pay attention to healthy competition between the two.

There is a significant mediating effect between the interaction effect of visual and auditory logos and consumer behavior between brand perception, credibility, and quality perception. In other words, if the visual and auditory logos released by the enterprise make consumers have a positive brand perception, credibility, and quality perception, then this perception will strengthen the positive influence connected with the interaction achieved of ocular and hearing logos impact consumer behavior, and further promote the improvement of sustainable development of enterprises. Businesses cultivate positive customer attitudes through audio–visual stimulation and media innovation. This study develops a model of visual and auditory intuitive associations with customer behavior utilizing the SOR model. It tests the reasonable model and checks the speculations proposed in the review. The visual and auditory interactive relationship between information integration, information synergy, mutual competition, and matching degree is summarized and contributed to by this study. It also suggests that consumer support and purchase intention impact consumer behavior in terms of how they perceive a brand’s credibility and quality. Highlights and results demonstrate that logos significantly influence consumer behavior.

## Figures and Tables

**Table 1:** Survey scale measuring the interaction between visuals and sounds in logos.

No.	Factors	Problem Description	Sources
SQ1	Information integration	The application of vision and sound in the logo and the message expressed are simple and intuitive	Pinto et al. [1,13,16,46]
SQ2		The application of vision and sound in the logo can accurately express the message of the brand	
SQ3		The application of vision and sound in the logo can express the connotation of the brand	
SQ4		The application of vision and sound in the logo can become an important label for the brand	
SQ5		The application of vision and sound in the logo presents a unique sensory experience to each other	
SQ6		The application of vision and sound in the logo is highly compatible	
SQ7	Information synergy	The application of vision and sound in the logo makes the presentation interesting	Climer et al. [13,16]
SQ8		The application of vision and sound in the logo makes the content colorful	
SQ9		The application of vision and sound in the logo is novel and trendy	
SQ10		The visual representation of the logo is too bright and eye-catching	
SQ11		The representation of sound in the logo is too loud and does not correspond to the sensory experience	
SQ12		The visual and auditory experiences in the logo are not synchronized	
SQ13	Mutual competition	The visuals and sounds in the logo can express the individuality of each	Kim et al. [19,37]
SQ14		The visuals and sounds in the logo can express their respective personalities	
SQ15		The visuals and sounds in the logo always have a clear purpose	
SQ16		The visual and sound expressions in the logo conflict with each other	
SQ17		The visual and sound in the logo are both overly distinctive and impactful	
SQ18		The visuals and sound in the logo are independent of each other and difficult to integrate	
SQ19	Information matching	The application of vision and sound in the logo is clear and precise	Grewal et al. [13,45]
SQ20		The application of vision and sound in the logo gives the impression that the brand is authoritative and reliable	
SQ21		The application of vision and sound in the logo makes people feel that the brand design is unique	
SQ22		The application of vision and sound in the logo is highly information matching	
SQ23		The application of vision and sound in the logo gives me a good experience and comfort	
SQ24		The combination of vision and sound in the logo provokes a desire for attention	

**Table 2:** Survey scale of consumer perception.

No.	Variables	Problem Description	Sources
OQ1	Brand perception	Brands that publish audio–visual ads are worth the money	Lafferty et al. [2,22,46]
OQ2		A good impression of brands that publish audio–visual ads	
OQ3		Brands that publish audio–visual ads can meet individual needs	
OQ4		Trust brands and their products that publish audio–visual advertising	
OQ5		Have a good impression of brands that publish audio–visual ads	
OQ6		Brands that publish audio–visual ads conform to a personal image	
OQ7	Quality perception	For enterprises that publish audio–visual advertisements, the quality of the products they produce is reliable	Zhou [52]
OQ8			
OQ9		Trust the quality of the products of companies that publish audio–visual advertisements	
OQ10		For enterprises that publish audio–visual advertisements, the quality of the products they produce is more reassuring	
OQ11	Consumer credibility	Enterprises that publish audio–visual advertisements are reliable	Zboja [51]
OQ12		Enterprises that publish audio–visual advertisements are trustworthy	
OQ13		Enterprises that publish audio–visual advertisements are reassuring	

**Table 3:** Survey scale of consumer behavior.

No.	Variables	Problem Description	Sources
RQ1	Consumer purchase intention	I am willing to purchase products and services from enterprises that publish audio-visual advertisements	Dodds and Grewal [8,13]
RQ2		I am willing to recommend the products of the enterprises that publish audio-visual advertisements to others	
RQ3		Enterprises that publish audio-visual advertisements are my first choice for related products	
RQ4		I will still prefer the enterprises that publish audio-visual advertisements when I make my next purchase	
RQ5	Consumer support	I will recommend the enterprises that publish audio-visual advertisements and their products to someone I know	Zeithaml [53]
RQ6		I will recommend the enterprises that publish audio-visual advertisements and their products to people who ask for my opinion	
RQ7		I will recommend the enterprises that publish audio-visual advertisements and their products to my friends and family	

**Table 4:** Cronbach’s α.

Variables	Statistical Analysis of Reliability		
	Cronbach’s α	Cronbach’s α Determined on Established Terms	Number of Items
Audio-visual interaction effect variables	0.893	0.894	24
Consumer behavior variables			
Brand perception variable	0.878	0.878	6
Credibility variable	0.833	0.833	3
Quality perception variable	0.859	0.859	4
Consumer perception varaibles			
Consumer purchase intention	0.842	0.842	4
Consumer support	0.819	0.819	3

**Table 5:** KMO and Bartlett’s test.

Variables	Bartlett Test of Sphericity			
	Kaiser–Meyer–Olkin Measure of Sampling Adequacy	Bartlett Test of Sphericity		
		Chi-Squared Approximation	Degree of Freedom	Significance
Audio-visual interaction effect variables	0.918	15,047.087	276	0
Brand perception variable	0.906	3703.438	15	0
Credibility variable	0.725	1612.06	3	0
Quality perception variable	0.824	2456.281	6	0
Consumer purchase intention	0.819	2175.793	6	0
Consumer support	0.718	1480.77	3	0

**Table 6:** Regression analysis of stimulus and response variables.

	Model 1	Model 2
	B	B
(Constant)	0.499	0.499
1. Your gender	0.944	0.944
2. Your age	−0.029	−0.029
4. Your education level	0.036	0.036
6. Your occupation	0.05	0.05
8. The area where you live for a long time belongs to	0.012	0.012
(Constant)	−0.499	−0.539
1. Your gender	0.621	0.618
2. Your age	−0.014	−0.013
4. Your education level	0.025	0.027
6. Your occupation	0.036	0.039
8. The area where you live for a long time belongs to	0.011	0.01
Interactive relationship of audio-visual logos	0.697	
information integration		0.138
Information synergy		0.257
Information competition		0.137
Information matching		0.176
R-squared	0.609	0.615
F	363.949	248.267
*p*	0	0

**Table 7:** Analysis of mediating effects.

	3rd Model	4th Model	5th Model	6th Model
	B	B	B	B
(Constant)	−0.499	−0.499	−0.499	−0.499
1. Your gender	0.621	0.621	0.621	0.621
2. Your age	−0.014	−0.014	−0.014	−0.014
4. Your education level	0.025	0.025	0.025	0.025
6. Your occupation	0.036	0.036	0.036	0.036
8. The area where you live for a long time belongs to	0.011	0.011	0.011	0.011
Interactive relationship of audio-visual logos	0.697	0.697	0.697	0.697
(Constant)	−0.662	−0.542	−0.567	−0.545
1. Your gender	0.565	0.612	0.597	0.605
2. Your age	−0.016	−0.015	−0.012	−0.017
4. Your education level	0.029	0.028	0.025	0.024
6. Your occupation	0.034	0.034	0.035	0.035
8. The area where you live for a long time belongs to	0.003	0.009	0.005	0.006
Interactive relationship of audio-visual logos	0.465	0.628	0.6	0.618
Consumer perception	0.355		0.15	0.116
Credibility		0.104		
Brand perception			0.15	
Quality perception				0.116
R-squared	0.653	0.620	0.631	0.625
F	376.892	325.727	341.057	331.451
*p*	0	0	0	0

## Data Availability

Not applicable.

## Supplementary Materials

The following supporting information can be downloaded at: https://www.mdpi.com/article/10.3390/bs13070613/s1, Figure S1: Confirmatory Factor Analysis (CFA); Figure S2: Structural equation modeling.
