# Supplementary figures and images for: A Study and Analysis of the Relationship between Visual—Auditory Logos and Consumer Behavior
Source: Behav Sci (Basel). 2023 Jul 24;13(7):613. doi: 10.3390/bs13070613 (PMC10376566; doi:10.3390/bs13070613)

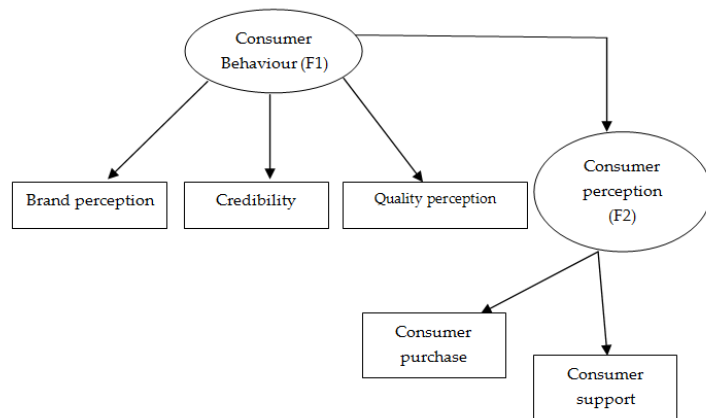

**Figure S1.** Confirmatory Factor Analysis (CFA).

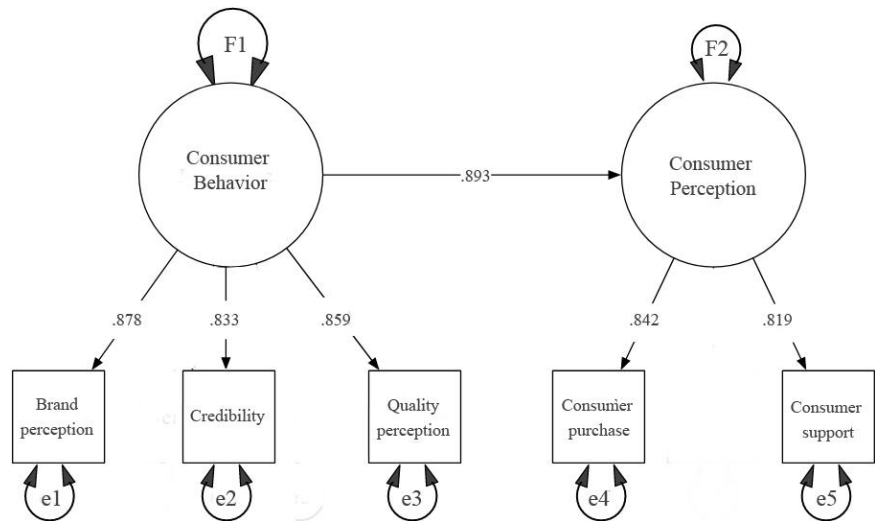

**Figure S2.** Structural equation modeling.

Supplement: Supplementary file 1 [file behavsci-13-00613-s001.zip › behavsci-2443072-Supplementary.pdf]
